# Association between frailty and pain in older people at high risk of future hospitalization

**DOI:** 10.3389/fpain.2025.1576691

**Published:** 2025-04-28

**Authors:** Huan-Ji Dong, Joakim Yang, Maria M. Johansson, Anneli Peolsson, Marco Barbero, Magnus Nord

**Affiliations:** ^1^ Department of Health, Medicine and Caring Sciences, and Pain and Rehabilitation Center, Linköping University, Linköping, Sweden; ^2^ Department of Activity and Health in Linköping, Linköping University, Linköping, Sweden; ^3^ Department of Health, Medicine and Caring Sciences, Linköping University, Linköping, Sweden; ^4^ Department of Health, Medicine and Caring Sciences, Unit of Physiotherapy, Linköping University, Linköping, Sweden; ^5^ Occupational and Environmental Medicine Centre, Department of Health, Medicine and Caring Sciences, Unit of Clinical Medicine, Linköping University, Linköping, Sweden; ^6^ Rehabilitation Research Laboratory 2rLab, Department of Business Economics, Health and Social Care, University of Applied Sciences and Arts of Southern Switzerland, Manno, Switzerland; ^7^ Primary Health Care Center Valla, Linköping, Sweden

**Keywords:** chronic pain, ADL, physical functioning, frailty, aging

## Abstract

**Background:**

Previous studies have demonstrated an independent association between pain and frailty, but knowledge about this association with different pain characteristics is limited.

**Objective:**

This study was embedded in a prospective, pragmatic, matched-control multicenter trial at 19 primary care practices in south-eastern Sweden (ClinicalTrials.gov 170608, ID: NCT03180606), aiming to investigate the association between frailty and pain characteristics among older people (75+) at high risk of hospitalization.

**Methods:**

High risk of hospitalization was identified using case-finding algorithm including 32 diagnostic codes of morbidities and healthcare use. Frailty was assessed by a nurse-physician team using Clinical Frailty Scale (*N* = 389). Data on pain aspects, physical and ADL functioning were collected in the self-reported questionnaires.

**Results:**

One in three (*n* = 133, 34%) was classified as frail. About 36% (*n* = 142) reported frequent pain (from several times per week to constantly). Slightly over 40% reported pain lasting longer than 3 months (*n* = 163, 41.9%) and/or having regional or widespread pain (*n* = 165, 42.4%). In comparison to non-frail peers, frail participants reported higher pain intensity, more ADL-dependency, less physical activity, and more anxiety/depression (*p* < 0.01). In logistic regression analysis, pain frequency [Odds Ratio (OR) 1.8, 95% confidence interval (CI): 1.2–2.8] was associated with frailty. However, the models with ADL-staircase score (OR: 1.4, 95% CI: 1.2–1.6) had a higher explanatory power (Nagelkerke *R*^2^: 0.39) in predicting frailty than those without this aspect (*R*^2^: 0.10 and 0.13).

**Conclusion:**

In older people at high risk of hospitalization, pain frequency seemed to be related to frailty, whilst ADL dependency demonstrated a stronger association.

## 1 Introduction

The aging population increases rapidly around the world due to longer life expectancy, medical development, and improvement of health care service. Most older people is healthy, but multimorbidity (including pain conditions) and frailty increase with age. Frailty is characterized by an age-related decline in function in various organ systems, leading to increased vulnerability to external stressors (1–3). This vulnerability leads to a reduction in a frail person's functional ability and a high risk of hospitalization (4, 5). In a previous interview study with a group of frail older adults, we found that many of them adapted to their health problems and functional decline but kept a strong desire to live independently. They expressed fear of pain and suffering as well as losing independence in the end-of-life (6).

The International Association for the Study of Pain (IASP) defines pain as “An unpleasant sensory and emotional experience associated with, or resembling that associated with, actual or potential tissue damage” (7). Pain is a personal experience and pain characteristics are often measured such as frequency, duration, intensity, localization and spreading (8). Chronic pain, referring to pain persisting or recurring at least three months, is viewed as a disease of its own defined by the biopsychological model of pain (9). It has a multidimensional aspect and interacts with biological, psychological, and social factors reciprocally (9). It is well-known that chronic pain is associated with a higher degree of mortality, morbidity, and healthcare consumption. The sufferings of chronic pain are also common in the old age, as many older people with chronic pain report disability, lower life satisfaction, and poorer health status in general (10).

Previous research demonstrated an independent association between pain and frailty, after accounting for comorbidities and psychosocial factors (11). However, knowledge about this association with pain characteristics is rather limited, and functional aspects have yet to be considered together with pain characteristics. The current study aimed to investigate the association between frailty and pain characteristics in older adults with a high risk of hospitalization. We also explored this relationship with consideration of physical functioning and psychological well-being.

## 2 Methods

### 2.1 Study population from an interventional study trial

**Figure 1:**
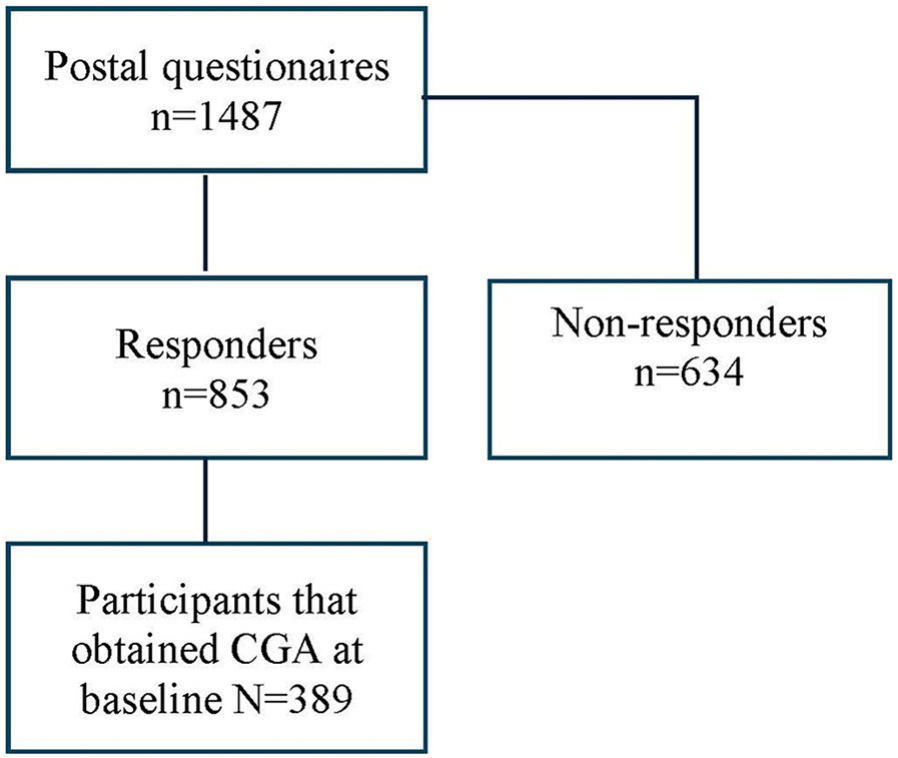
Study population.

This study was embedded in a prospective, pragmatic, matched-control multicenter trial at 19 primary care practices in south-eastern Sweden (12). In March 2017, we identified 1,604 individuals aged 75 years older with a high risk of hospitalization using case-finding algorithm including 32 diagnostic codes of morbidities related to unplanned admission to hospital (13). The trial included Intervention Group and Control Group at baseline, 1-year and 2-year follow-ups (Trial registration: ClinicalTrials.gov 170608, ID: NCT03180606) (14). All participants were invited to complete the postal questionnaires covering pain aspects and other health conditions on up to three occasions. The proactive healthcare in Intervention Group, led by patients' responsible general practitioner (GP), consists of a health assessment using the standardized evaluation form—the Primary Care Assessment Tool for Elderly (PASTEL) (15), to identify the health-related problems in need of medical treatment, rehabilitation, or prevention. PASTEL is constructed for the project and is based on the holistic approach of Comprehensive Geriatric Assessment (CGA) (15). PASTEL combines the diagnostic and therapeutic purposes, covering medical, functional, psychological, and social domains. An overview of patients' medical history including an evaluation of current medication use as well as physical measures were included in the clinical assessment. The GPs and nurses were introduced to the CGA tool, Frailty assessment with the Clinical Frailty Scale and the process of intervention prior to the study. For Intervention Group, this individual pragmatic assessment led to further rehabilitation and prevention such as home-based exercise program, prescription of assistance technology (e.g., walker, adapted toilet, bath/shower technology) and the extent to facilitating assistance (i.e., transportation service, community assistance, and personal alarm) in everyday life when needed. CGA was not included in usual care for Control Group. A postal questionnaire was delivered to all participants at three times during the study period for both groups. The current study population was restricted to all participants who received Comprehensive Geriatric Assessment (CGA) at baseline year (*N* = 389) and answered the first postal questionnaire (Figure 1). The study was approved by the regional ethical review board in Linköping (Dnr: 2016/347-31). All participants filled in an informal consent.

### 2.2 Measures

#### 2.2.1 Socio-demographic factors

The information on age and sex of one person were from Statistics Sweden (SCB). Highest education level (no education, less than nine years of compulsory school or similar, nine years compulsory school or similar, secondary school, or university/college), living situation (living alone, with cohabitant or with children) and marital status (married/co-habited and unmarried/widow) were asked in the postal questionnaire.

#### 2.2.2 Pain characteristics: frequency, duration, intensity and extent

Pain frequency was measured by letting patients assess how often they experience pain, with the scale ranging from 1 to 5: 1 = “never”; 2 = “occasionally”; 3 = “once per day”; 4 = “several times per day”; and 5 = “constantly”.

Pain duration was measured by asking “How long have you been suffering from pain?”. The answer was given by the number of months. The answer was also recorded as chronic pain (lasting longer than 3 months) and no chronic pain.

Pain intensity was measured by a visual analog scale reflecting pain over the preceding seven days (VAS Pain–7d). Scores are made by marking a 100 mm line, with 0 mm meaning no pain and 100 mm = worst imaginable pain.

Pain extent was used to identify pain localization and spreading, quantified through pain drawings. Participants provided information about their pain location by coloring two body charts attached in the postal questionnaire. One displayed a frontal and the other a ventral view of the body. All drawings were further digitalized and processed by calculating the total number of pixels in each drawing (1,024 × 78 pixel) (16, 17). Furthermore, using the manikin, local pain was generated when it was marked on just one section (or two sections when sections were equally marked on the front and back of e.g., hip, knee, shoulder, or arm). Widespread pain was pain marked in at least two sections in two contralateral limbs and the axial skeleton and marked equally on the front and on the back of the body. Regional pain was defined as pain shaded on the manikin that did not meet the criteria for the other two categories (18, 19).

#### 2.2.3 Frailty

The process of measuring frailty was divided into three processes (12, 20). First, an interview with multiple-choice questions and self-rating health was conducted by a nurse using the PASTEL tool (15). The second part consisted of a physical examination, a review of medical records and an evaluation of the patient's thoughts about current and future healthcare needs. A third step was that the medical team (GP together with the nurse) made an estimation of frailty using Clinical Frailty Scale (CFS) (21–23). CFS has a scoring system from 1 (very fit) to 9 (terminally ill) and it is complimented by a visual chart to assist in the classification of frailty. Higher scores in CFS indicate an increased risk of mortality and morbidity. The original CFS has high reliability and validity (21, 23). Based on the skewed distribution of the number of participants assessed over the different frailty scores (from 1 to 9), CFS was also categorized to non-frail category (scoring 0–4) and frail category (scoring 5–9) (22, 23).

#### 2.2.4 Anxiety/depression

EQ-5D-3L is a generic measurement for health-related quality of life and consists of five dimensions: mobility, self-care, usual activities, pain/discomfort, and anxiety/depression (24). Each dimension can be answered with the three following levels: no problems (level 1), some problems (level 2), and extreme problems (level 3). In this study, we used the dimension of anxiety/depression to measure the perceived anxiety/depression.

#### 2.2.5 Physical functioning

In the postal questionnaire, the participants were asked about their daily physical activity (e.g., walking or bicycling to the store, going out with dogs, gardening, shoveling snow or some other activity) and regular physical exercise (exercise/sport/open-air activity on a week basis, but not included in the everyday life activities above) during the past 6 months. A combination of the answers to both questions generated the physical activity level 1–4: 1 = inactivity, 2 = low activity, 3 = moderate activity, and 4 = high activity (25, 26). Good internal consistency (Cronbach's alpha of 0.79) was demonstrated in the present study.

Activity of Daily Living (ADL) was measured by the extended version of KatźADL index (ADL-staircase) (27). ADL-staircase covers both Personal ADL (PADL) and Instrumental ADL (IADL), containing the following 10 activities: feeding, cleaning, bathing, toilet visits, dressing, shopping, transportation, feeding, continence, and transfer. Each activity is graded by three levels (1 = independent, 2 = partially dependent, and 3 = dependent) and the total score is summarized into a scale from 10 to 30, where higher scores indicate greater dependency. Good construct validity has been reported previously (28). Good internal consistency (Cronbach's alpha of 0.86) was demonstrated in the present study.

### 2.3 Statistical analysis

Statistical analyses were conducted in IBM SPSS statistics (version 29.0. NY, USA). Data are reported as mean with standard deviations (SD), median with interquartile ranges (IQR) and as numbers with percentages (%) where appropriate. *χ*^2^-test for comparison between different categorical variables and Mann–Whitney *U* test for non-normally distributed variables were performed. A *p*-value under 0.05 was regarded as statistically significant. Effect size (ES) calculation for within-group analysis were computed using a calculator when appropriate (Phi-value for the Pearson *χ*^2^-test and rank-biserial correlation r for Mann–Whitney *U* test). Small ES is considered when Phi or *r* = 0.10–0.29, medium ES when Phi or *r* = 0.30–0.49, and large ES when phi or *r* = 0.50 and higher values (29). Correlation analysis between CFS grading and variables of interest was performed using Spearman's Rho correlation test.

To explore the odds of frailty (CFS scoring ≥5), binary logistic regression analysis (Forward, likelihood ratio) was performed to explore the odds of frailty. In comparison to Backward selections, we chose an exploratory approach due to lack of previous evidence. Results were displayed as odds ratio (OR) and 95% confidence interval (CI 95%). Firstly, univariate analysis was performed first for each predictor. A lax criterion with *p*-value <0.25 was used to select candidate variables due to limited prior studies in this field (30). Then, three multivariate regression models were performed by selecting (Forward, likelihood) pain characteristics (Model 1), psychological aspect (Model 2), and physical functioning (Model 3). Sociodemographic factors were selected as confounding factors to be entered in the models based on prior knowledge (31–34). We further examined collinearity among the categorical variables using the phi (Φ, Φ ≥ 0.30 indicating high correlation) (35). Due to the collinearity between marital status and living situation, we selected marital status as it showed significance regarding frailty status (Table 1). The Hosmer and Lemeshow test was used to examine the goodness of fit (*p* > 0.05). Logistic regression analyses use listwise deletion to handle the missing cases. To examine if missing data can lead to biased results, we also performed a sensitivity analysis where we used multiple imputation to handle the missing data as a comparison to the results based on “real completed cases” (see Supplementary Material).

**Table 1:** Comparison of different factors between individuals with and without frailty. Results are reported as number of participants (%) unless otherwise stated.

Characteristics	Total *n* = 389	Non-frail *n* = 256	Frail *n* = 133	*P*-value	ES (phi or *r*)
Age, *n* (%)					
75–84	224 (58)	154 (60)	70 (53)	0.16	0.07
85+	165 (42)	102 (40)	63 (47)		
Women, *n* (%)	195 (51)	125 (49)	70 (53)	0.48	0.04
Education, *n* (%) (*n* = 249)					
Less than or 9 years	126 (51)	95 (53)	31 (44)	0.21	0.79
Over 9 years	123 (49)	84 (47)	39 (56)		
Marital status, *n* (%) (*n* = 255)					
Married/co-habited	134 (53)	88 (47)	23 (33)	0.006	0.17
Unmarried/Widow	121 (47)	98 (53)	46 (67)		
Living situation, *n* (%) (*n* = 258)					
Living alone	123 (48)	93 (50)	30 (42)	0.23	0.08
With a partner or children	135 (52)	93 (50)	42 (58)		
Pain frequency, *n* (%) (*n* = 252)					
Never/occasionally	110 (44)	85 (47)	25 (36)	0.07	0.20
Sometime every day	88 (35)	68 (37)	20 (29)		
Several times every day/Constant daily	54 (21)	30 (16)	24 (35)		
Pain intensity-VAS (*n* = 223)					
Median (IQR)	41 (20.5–61.5)	37 (16.5–57.5)	53.5 (31.5–75.5)	0.004	0.19
Pain duration, month (*n* = 175)					
Median (IQR)	20 (10–48)	20 (10–48)	19 (9.3–48)	0.97	0.002
Over 3 months	163 (93)	120 (93)	43 (93.5)	0.92	0.008
Pain extent, percentage (*n* = 256)					
Median (IQR)	2.02 (0.58–5.44)	1.90 (0.58–4.90)	2.11 (0.67–7.32)	0.44	0.08
No pain	59 (23)	42 (22.8)	17 (23.6)	0.92	0.04
Local pain	32 (12.5)	22 (12)	10 (13.9)		
Regional pain	101 (39.5)	72 (39.1)	29 (40.3)		
Widespread pain	64 (25)	48 (26.1)	16 (22.2)		
EQ-5D Anxiety/depression, *n* (%) (*n* = 241)					
No problem	122 (48)	99 (54)	23 (33)	0.007	0.2
Moderate	123 (49)	80 (44)	43 (61)		
Severe problem	8 (3)	4 (2)	4 (6)		
Physical activity, *n* (%) (*n* = 173)					
Inactivity	50 (29)	26 (22)	24 (45)	0.003	0.28
Low activity	37 (21)	24 (20)	13 (25)		
Moderate activity	74 (43)	59 (49)	15 (28)		
High activity	12 (7)	11 (9)	1 (2)		
ADL-stair case score (*n* = 258)					
Median (IQR)	12 (9.5–14.5)	11 (9.5–12.5)	16 (13–19)	<0.001	0.48

ES, effect size; IQR, interquartile range; VAS, visual analog scale; ADL, activities of daily living.

## 3 Results

**Figure 2:**
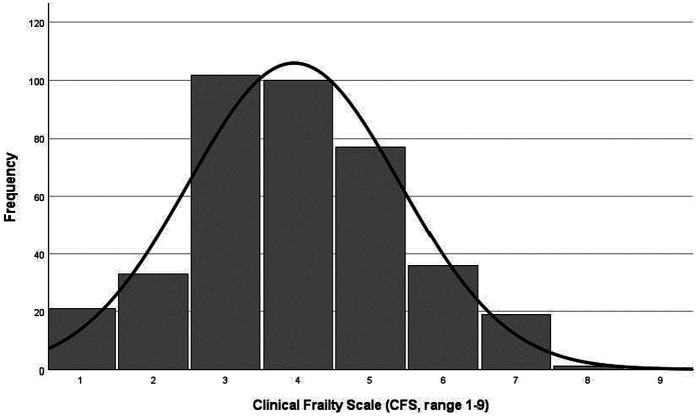
Distribution of CFS score.

Mean age was 83.45 ± 5.09 years and 51% were female in this study population (Table 1). Mean CFS score was 3.95 ± 1.46, and approximately every other participant was classified as less vital (102, 26.2%) or vulnerable (100, 25.7%, Figure 2). Over one-third was detected as having frailty (CFS ≥ 5, 133, 34%), with almost equal distribution in both sexes (49% of men and 51% of women, respectively).

As shown in Table 1, approximately half of the participants completed high school or higher education, and no significant difference was found between Frailty Group (FG) and Non-Frailty Group (NFG). More participants in FG (67%) were unmarried or widowed than NFG participants (53%, *p* = 0.006, small ES). The likelihood of living alone or living with others (partner and/or children) was similar regardless of frailty status (approximately 50%, *p* > 0.05).

With non-responded participants (missing values) included, more than one in three (142, 36.5%) reported frequent pain (sometime every day, several times every day, and constantly daily), slightly over 40% had pain lasting longer than 3 months (163, 41.9%), and regional or widespread pain (165, 42.4%). It was extremely common to report chronic pain among the valid responses (163, 93%). Significant difference between the groups was found in pain intensity, where it was higher in FG (Median 53.5, IQR 31.5–75.5) than that in NFG (Median 37, IQR 16.5–57.5, *p* = 0.004, small ES).

FG participants were more likely than NFG peers to report severe anxiety/depression (*p* = 0.007, small ES). Regarding physical functioning, significantly lower physical activity level was showed in FG compared to NFG (*p* = 0.003, small ES). Higher scores of ADL-staircase were found in FG (Medan 16, IQR 13–19) compared to NFG (Median 11, IQR 9.5–12.5, *p* < 0.001, medium ES), indicating more ADL-dependency.

CFS grading was significantly correlated to most pain characteristics, psychological aspect and physical functioning (rho: 0.14–0.59, Table 2). Pain intensity showed significant correlations to all variables of interest. Pain frequency, EQ-5D anxiety/depression and score of ADL-staircase were correlated to all other variables except pain duration. Pain duration was only correlated to two other pain aspects (pain intensity and pain extent, *p* < 0.05).

**Table 2:** Correlations between CFS grading and independent variables.

	CFS	Pain frequency	Pain intensity	Pain extent	Pain duration	EQ-5D Anxiety depression	Physical activity
Pain frequency	0.20**	1					
Pain intensity-VAS	0.19**	0.65**	1				
Pain extent	0.14*	0.57**	0.48**	1			
Pain duration	0.03	0.13	0.18*	0.22**	1		
EQ-5D Anxiety depression	0.24**	0.22**	0.27**	0.23**	−0.02	1	
Physical activity	−0.47**	−0.23**	−0.22**	−0.05	−0.15	−0.10	1
ADL-Staircase	0.59**	0.26**	0.22**	0.18**	0.11	0.29**	−0.41**

CFS, clinical frailty scale; VAS, visual analog scale; ADL, activities of daily living.

* *p* < 0.05. **p < 0.01.

Univariate logistic analysis showed all pain characteristics except pain duration were significantly associated to frailty (Table 3). In multivariate logistic regression analysis, pain frequency (OR: 1.79, 95% CI: 1.15–2.77) was significantly associated with frailty (Table 3, Model 1). The association decreased to a marginal insignificance (*p* = 0.052) when anxiety/depression was tested into the model (Table 3, Model 2). When the score of ADL-staircase was considered in the model fitting, neither pain frequency nor anxiety/depression remained to show significant associations (Table 3, Model 3). This model with ADL-staircase score (OR: 1.39, 95% CI: 1.22–1.58) had a higher explanatory power (Nagelkerke *R*^2^: 0.39) in predicting frailty than those without this aspect (*R*^2^: 0.10 and 0.13). The final model 3 was also examined by including one pain variable (frequency, intensity, extent and duration) at a time (Logistic regression, Enter). None of the pain characteristics showed significant association with frailty (see Supplementary Material).

**Table 3:** Result of multivariate logistic regression (forward LR) model. OR (95% CI). Frailty (CFS score ≥ 5) was the dependent variable of interest.

Independent variables	Regression models			
	Univariate analysis	Model 1	Model 2	Model 3
Pain characteristics				
Pain frequency	1.60 (1.12–2.29)**	1.79 (1.15–2.77)*	1.56 (1.00–2.46)	EXCL
Pain intensity-VAS	1.02 (1.01–1.03)**	EXCL	EXCL	EXCL
Pain extent	1.04 (1.01–1.08)*	EXCL	EXCL	EXCL
Pain duration	1.00 (0.99–1.01)	NA	NA	NA
Psychological aspect				
EQ-5D anxiety/depression	2.23 (1.34–3.69)**	NA	2.12 (1.15–3.93)**	EXCL
Physical functioning				
Physical activity level	0.51 (0.35–0.73)**	NA	NA	EXCL
Score of ADL-staircase	1.39 (1.27–1.53)**	NA	NA	1.39 (1.22–1.58)**
Nagelkerke *R*^2^	0.03–0.35	0.10	0.13	0.39
*N*	173–258	218	213	148

EXCL, excluded variable; NA, not applicable; OR, odds ratio; 95% CI, 95% confidence interval. Socio-demographic factors (age, sex, education level and marital status) were adjusted for Model 1–3.

* *p* < 0.05.**p < 0.01.

## 4 Discussion

The main finding of this study is that pain characteristics (frequency, intensity, extent, and duration) were not associated with the severity of frailty in an aging population with high risk of future hospitalization. Once pain frequency was included in the logistic regression model after accounting for psychological aspect and physical functioning, the other pain characteristics did not contribute additional information. In comparison, ADL dependency had a stronger association with frailty than pain characteristics and psychological aspect (anxiety/depression).

It is hypothesized that the mechanism on pain-frailty relationship is that pain, whether chronic, severe, or widespread, may infringe on different physiological systems leading to decreased ability to perform physical activities (36). Regarding pain as an impact on physical function, researchers found pain contributed to a limitation in movement, fatigue, and lower nutritional intake (37). The experience of pain potentially creates a state of vulnerability to stressors, which may explain why individuals with pain are more prone to develop frailty and experience worsening frailty (36, 38). Pain, particularly chronic pain, has also been linked to a higher incidence of anxiety and depression (38). This in turn is more likely to lead to different consequences such as a sedentary lifestyle, malnutrition, and weight loss, which are known prerequisites for frailty development (39).

To our knowledge, few studies have investigated different pain characteristics and the associations to frailty in aging populations. According to a systematic review on the relationship between chronic pain and frailty among community-dwelling older adults, previous studies mostly focused on investigating how chronic pain influences frailty, but not considering pain intensity, frequency, and extent (11). Another study concluded that chronic pain, rather than pain intensity is positively associated with the grading of frailty in older hospitalized patients (40). This reinforces the idea that the main determinant of frailty is chronicity of pain. Moreover, the study found that widespread pain (vs. localized pain) predicted a higher grading of frailty. Comparatively, pain extent measured by pain drawings was not significantly associated with frailty in our study, when pain frequency was considered in the regression analysis. A clear difference from the above-mentioned studies is that our study population can be classified as a vulnerable group. For example, we noted an extremely high prevalence of chronic pain among the valid responses (93%), which is much higher than community-dwelled populations.

Without accounting for physical functioning, psychological problem (anxiety/depression) was a significant predictor to frailty. The results showed that the odds of being frail was 2.12 times higher for each level increase in anxiety or depression measured by EQ-5D (Table 3, Model 2). This finding was in agreement with literature (41). The significant effect, however, disappeared after the score of ADL-staircase was considered in the analysis (Table 3, Model 3). To be noted, perceived anxiety or depression should not be regarded equally as clinical anxiety or depressive symptoms that usually included clinical instruments in measurement. We speculate that the self-perceived psychological well-being might have a less impact on frailty than that was measured by clinical instruments (41).

In terms of ADL-dependency, we found that ADL-staircase score was a significant predictor for frailty. This finding can also be confirmed by earlier studies (42, 43). As individuals experienced difficulties in performing ADLs, they became more susceptible to physical and cognitive decline, which could ultimately result in frailty (42). Managing ADLs is essential for maintaining independence and quality of life, and impairment can lead to a loss of functionality and an increased risk of adverse health outcomes. To date, ADL assessment is included in frailty screening for assessing physical function and for identifying individuals who are at risk of functional decline. A systematic literature search assessing ADL in frailty instruments found it unclear whether disability in ADL should be considered an outcome of frailty, a characteristic of frailty, or a predictor of frailty (43). This inconclusiveness reflects the heterogeneity of the way frailty and ADL disability are defined and the subsequent different approaches toward the interrelationship between frailty and ADL disability. That study highlighted the need for a more comprehensive and standardized definition of the specific ADLs to be included in the frailty instruments.

From a pain rehabilitation perspective, it is reasonable to address rehabilitation interventions on improving, delaying or preventing deterioration of functional status and ADL ability, which also matches one of the main goals of proactive healthcare. Our clinical practice may shift from merely prescribing pain medications to rehabilitation approaches. It is well acknowledged that multiple pharmacologic agents might not always be of help (44, 45). Non-pharmacological approaches, such as referrals to the rehabilitation team (physiotherapist, occupational therapists, dieticians, etc.) are supposed to be included in the team-based interventions for pain management among vulnerable older people. With this knowledge, health professionals may also challenge themselves in motivating and supporting vulnerable older people in pain rehabilitation and everyday life, and in enhancing independence at home (46). More studies on the effectiveness of different rehabilitation interventions in older adults at risk of frailty and functional decline are also needed.

### 4.1 Strengths and limitations

To our knowledge, this is the first study to investigate the association between different pain characteristics and frailty among older people with a risk of future hospitalization. As the population also was part of a pragmatic intervention study in primary care, it is a highly relevant group to study from a clinical perspective. The instruments used to measure frailty and pain extent are well validated (17, 21, 24, 28).

Some limitations need to be considered. Firstly, the data collected in this study are embedded in a larger trial and the questionnaires were not specifically related to the aim of this study, which could have contributed to a high number of nonresponses to pain-related questions. Individuals might be more likely to complete questions related to their current health state and skip others they thought were irrelevant. For example, a minority of the participants (*n* = 36, 9.3%) did not answer any question of pain aspects. It is difficult for us to distinguish no pain complaint from missing values (left the question blank). A second limitation is response bias, due to a minority of the responses being provided by close relatives or caregivers (proxy). Some items (e.g., pain extent measured by pain drawing), could have excluded older people with impaired cognitive capacity if they could not get help from proxy. Thirdly, the cross-sectional study design limits the interpretation of causal-effect relationship. We only analyzed the relationship between different pain characteristics and frailty at one time point. Our results cannot draw any conclusion on factors associated with onset and/or development of frailty (36).

### 4.2 Conclusion

In older adults (75+) with a high risk for future hospitalization, pain frequency seemed to be related to frailty (CFS ≥ 5). When psychological aspect and physical functioning were considered, ADL dependency showed a stronger association with frailty than pain frequency. The study findings suggest that pain frequency and ADL dependency should be recognized as pertinent health issues to be addressed in pain rehabilitation to identify frailty in vulnerable older adults. Enhancing ADL functioning can be a realistic goal in pain rehabilitation as this aspect showed a strong association with frailty.

## Data Availability

The raw data supporting the conclusions of this article will be made available by the authors, without undue reservation.
