# Supplementary material for: Association between frailty and pain in older people at high risk of future hospitalization
Source: Front Pain Res (Lausanne). 2025 Apr 28;6:1576691. doi: 10.3389/fpain.2025.1576691 (PMC12083458; doi:10.3389/fpain.2025.1576691)
Supplement: Supplementary file 1 [file Datasheet1.pdf]

Association between Frailty and Pain in Older People at High Risk of Future Hospitalization

Supplemental document:

i. Analysis of missing data: multiple imputation

Missing data are often categorized into the following three types: missing completely at random (MCAR), missing at random (MAR), and missing not at random (MNAR) [1]. Traditional statistical analysis such as linear regression is based on the assumption of MCAR. However, most clinical epidemiological research is neither MCAR nor MNAR but MAR [2]. Multiple imputation (MI) can be used to handle missing data under the MAR assumption, with a purpose of providing unbiased and valid estimates of associations based on information from the available data.[1, 3] As described by Harel et al, MI consisted of 3 steps: data imputation (i.e. multiple imputation by chained equations (MICE)), analysis of each imputed data set (i.e. linear regression) and a final estimate of coefficients in the combination of all the imputed data sets (pooled) [3].

Before multiple imputations, we performed the analysis of missing data patterns. STable 1 showed the number of missing cases in each variable of interest. A total of 863 values (24.3%) were missing and 256 (79.3%) of the 389 cases contained at least one missing value. Multiple imputation was applied using m =100 (iterations =50) imputed data set with predictive mean matching method and included all the variables of interest (predictors and outcomes). Multiple imputed datasets for each model were generated in SPSS IBM SPSS statistics (version 29.0. NY, USA.). Several arguments and suggestions on the number of imputations have been discussed previously [2, 4, 5]. The separate analysis (original dataset and 100 multiple imputed datasets) was performed first and followed by the final analysis with the pooled sample. Using the pooled sample, we found that our models did not in fact change much as a result of imputing missing data. As shown in STable 2, there were similar significant associations between variables of interest and being frail (CFS score ≥5). In Model 2, the association of pain frequency and frailty disappeared ($p = 0.061$). The 95% confidence was slightly larger when missing cases were considered in the regression models.

ii. To examine one pain characteristic at a time (frequency, intensity, extent and duration) associated with frailty

The final model 3 (see original paper) was also examined by including one variable of pain (frequency, intensity, extent and duration) at a time (Logistic regression, Enter). Logistic regression analysis method (Forward, likelihood ratio) was then used to examine psychological aspect and physical functioning. As shown in STable 3, none of the pain characteristics alone remained in the models, but the variables of physical functioning showed significant association with frailty.

References

1. Rubin DB: **Inference and missing data**. *Biometrika* 1976, **63**(3):581-592.
2. Pedersen AB, Mikkelsen EM, Cronin-Fenton D, Kristensen NR, Pham TM, Pedersen L, Petersen I: **Missing data and multiple imputation in clinical epidemiological research**. *Clin Epidemiol* 2017, **9**:157-166.
3. Harel O, Mitchell EM, Perkins NJ, Cole SR, Tchetgen Tchetgen EJ, Sun B, Schisterman EF: **Multiple Imputation for Incomplete Data in Epidemiologic Studies**. *American Journal of Epidemiology* 2017, **187**(3):576-584.
4. Stuart EA, Azur M, Frangakis C, Leaf P: **Multiple imputation with large data sets: a case study of the Children's Mental Health Initiative**. *American journal of epidemiology* 2009, **169**(9):1133-1139.
5. Heymans MWE, Iris.: **Applied Missing Data analysis Book with SPSS and (R) studio**. In. Amsterdam: Heymans and Eekhout; 2019.

Supplementary Table 1. Missing cases in each variable of interest considered in regression models.

	Missing		Valid N
	N	Percent	

Physical activity level	216	55.5%	173
Pain frequency	195	50.1%	194
Pain intensity-VAS	166	42.7%	223
Education level	140	36.0%	249
EQ-5D-anxiety/depression	136	35.0%	253
Marital status	134	34.4%	255
Pain extent	133	34.2%	256
Score of ADL-staircase	131	33.7%	258
Gender	0	0.0%	389
Age	0	0.0%	389
CFS grading	0	0.0%	389

Supplementary Table 2. Association between CFS (CFS ≥5) and pain characteristics.

	**Model 1**	**Model 2**	**Model 3**
**Pain characteristics**			
**Pain frequency**	1.60 (1.08-2.36) *	1.44 (0.98-2.12)	NA
**Psychological aspect**			
**EQ-5D anxiety/depression**	NA	2.28 (1.37-3.79) **	NA
**Physical functioning**			
**Score of ADL-staircase**	NA	NA	1.40 (1.22-1.60) **
**Nagelkerke R^2 (m=100)**	0.06-0.19	0.10-0.27	0.20-0.58
**N**	389	389	389

* $p < 0.05$. ** $p < 0.01$. NA: not applicable. Socio-demographic factors (age, sex, education level and marital status) were adjusted for Model 1-3. OR: odds ratio. 95% CI: 95% confidence interval.

Supplementary Table 3. Association between CFS (CFS ⩾5) and each pain variable at a time included.

	Model with Pain frequency	Model with Pain intensity	Model with Pain extent	Model with Pain duration
**Pain characteristics**				
**Pain frequency**	0.96 (0.54-1.71)	NA	NA	NA
**Pain intensity -VAS**	NA	1.01 (0.99-1.03)	NA	NA
**Pain extent**	NA	NA	0.74 (0.50-1.08)	NA
**Pain duration**	NA	NA	NA	1.001 (0.999-1.002)
**Psychological aspect**				
**EQ-5D anxiety/depression**	EXCL	EXCL	EXCL	EXCL
**Physical functioning**				
**Physical activity level**	0.65 (0.41-1.03)	EXCL	0.64 (0.41-1.01)	0.63 (0.40-0.99)*
**Score of ADL-staircase**	1.31 (1.16-1.47) **	1.36 (1.19-1.56) **	1.32 (1.17-1.49) **	1.30(1.16-1.46) **
**Nagelkerke *R*2**	0.38	0.40	0.40	0.40
**N**	162	145	166	166

* $p < 0.05$. ** $p < 0.01$. EXCL: excluded variable from the model after regression analysis (forward likelihood ratio); NA: not applicable in the current model building; OR: odds ratio. 95% CI: 95% confidence interval. Socio-demographic factors (age, sex, education level and marital status) were adjusted.
